# Aberrant expression of miR-153 is associated with overexpression of hypoxia-inducible factor-1α in refractory epilepsy

**DOI:** 10.1038/srep32091

**Published:** 2016-08-24

**Authors:** Yaohua Li, Cheng Huang, Peimin Feng, Yanping Jiang, Wei Wang, Dong Zhou, Lei Chen

**Affiliations:** 1 Department of Neurology, West China Hospital of Sichuan University, Chengdu, 610041, Sichuan, People’s Republic of China; 2 Department of integrated traditional and western medicine, Hospital of Chengdu University of Traditional Chinese Medicine, Chengdu, 610075, Sichuan, People’s Republic of China

## Abstract

Evidence suggest that overexpression of hypoxia-inducible factor-1α (HIF-1α) is linked to multidrug resistance of epilepsy. Here we explored whether aberrant expression of HIF-1α is regulated by miRNAs. Genome-wide microRNA expression profiling was performed on temporal cortex resected from mesial temporal lobe epilepsy (mTLE) patients and age-matched controls. miRNAs that are putative regulator of HIF-1α were predicted via target scan and confirmed by real-time quantitative polymerase chain reaction (RT-qPCR). Mimics or miRNA morpholino inhibitors were transfected in astrocytes and luciferase reporter assay was applied to detect HIF-11α expression. Microarray profiling identified down-regulated miR-153 as a putative regulator of HIF-1α in temporal cortex resected from surgical mTLE patients. RT-qPCR confirmed down-regulation of miR-153 in plasma of mTLE patients in an independent validation cohort. Knockdown of miR-153 significantly enhanced expression of HIF-1α while forced expression of miR-153 dramatically inhibited HIF-1α expression in pharmacoresistant astrocyte model. Luciferase assay established that miR-153 might inhibit HIF-1α expression via directly targeting two binding sites in the 3′UTR region of HIF-1α transcript. These data suggest that down-regulation of miR-153 may contribute to enhanced expression of HIF-1α in mTLE and serve as a novel biomarker and treatment target for epilepsy.

Epilepsy is one of the most prevalent neurological disorders worldwide that affects about 1.5–2% of the world population. It has been shown that 30–40% patients with epilepsy are pharmacoresistant to multiple anti-epileptic drugs (AEDs)^1^,^2^,^3^. Mesial temporal lobe epilepsy (mTLE), a special form of epilepsy, is particularly relevant due to its high frequency of therapeutic resistance. However, the molecular mechanisms underlying multidrug resistance of epilepsy remain largely unknown^4^,^5^,^6^.

Hypoxia-inducible factor 1a (HIF-1a) is the main transcription factor responsible for the cellular adaptation to hypoxia^7^. Recently, growing evidence indicates HIF-1a signaling is involved in various physiological processes in the brain^8^,^9^,^10^ as well as pathogenesis of various neurological diseases such as ischemic and hypoxic encephalopathy^11^. Activated HIF-1 is shown to transcriptionally enhance the expression of multidrug transporters such as P-glycoprotein (P-gp) in astrocytes, resulting in decreased accumulation of AED in the brain^12^. Consistent with these notions, we previously found that expression levels of HIF-1α and P-gp are coordinately elevated in hippocampus and temporal lobe of patients with mTLE as well as pharmacoresistant TLE rat model kindled by coriaria lactone^13^,^14^,^15^,^16^. Accordingly, targeting HIF-1α represents an attractive strategy to enhance the efficacy of current therapies of refractory epilepsy.

However, how HIF-1α expression is regulated during epileptogenesis and drug resistance formation remains largely unknown. MicroRNAs (miRNAs) are small, noncoding RNA species that play critical roles in regulating protein expression via inhibiting translation and/or mRNA degradation^17^. Previous studies suggest that HIF-1α expression is tightly regulated by multiple miRNAs in different tissues/cells. For example, it has been shown that miR-20b and miR-199a may regulate HIF-1α expression in MCF-7 breast cancer cells^18^ and cardiomyocytes^19^, respectively. Notably, recent study suggested that expression levels of numerous miRNAs are significantly dysregulated after status epilepticus in rat^20^. This raises the possibility that abnormally expressed miRNAs which contribute to the pathogenesis of epilepsy and enhanced expression of HIF-1α could be, at least in part, caused by loss of expression of some regulatory miRNAs.

To examine the possible role of miRNA in refractory epilepsy, we performed a genome-wide miRNA expression profiling studies to identify aberrantly expressed miRNAs in temporal cortex tissues isolated from mTLE patients. Via target prediction, we screened for significantly down-regulated miRNAs that are putative regulatory of HIF-1α expression. We then validated aberrant expression of these miRNAs in a larger validation cohort, and used functional assays to investigate their roles in regulating HIF-1α expression. These findings have largely extended the current concepts of mTLE pathogenesis to miRNA-mediated gene expression regulation.

## Results

### miR-153 is down-regulated in temporal cortex of mTLE patients

**Figure 1:**
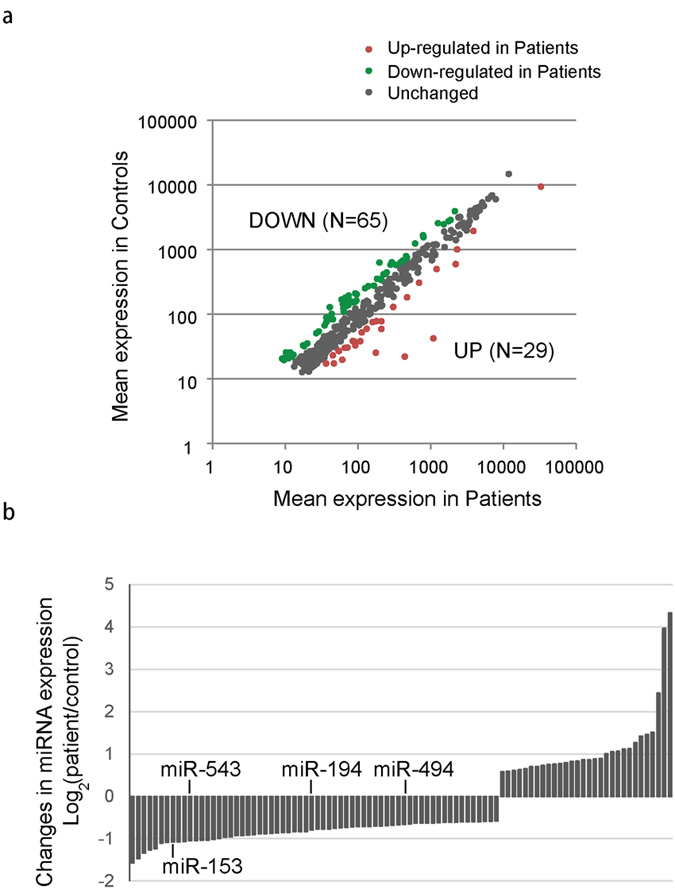
miRNA expression profiles of temporal lobe cortex resected from mTLE patients or controls. (**a**) Correlation of miRNA expression between mTLE patients and controls. Fold change >1.5 was considered as significant different. (**b**) Aberrantly expressed miRNAs were shown in the histogram. Down-regulated miRNAs that might be putative regulator of HIF-1α were highlighted.

**Figure 2:**
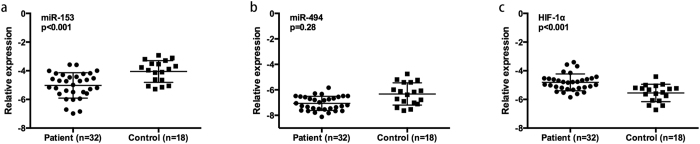
Relative expression of miR-153, miR-494 and HIF-1α in temporal cortex of 32 mTLE patients and 18 controls. (**a**) miR-153 was detected by quantitative realtime PCR in 32 mTLE cases and 18 matched controls. Differences in miRNA expression was determined by student’s t-test. (**b**) miR-494 expression in mTLE and controls. (**c**) HIF-1α expression in mTLE and controls.

As shown in Fig. 1a, of all 428 miRNA expressed in temporal cortex, 65 miRNAs were down-regulated and 29 miRNAs were up-regulated in mTLE patients with a fold change >1.5 (see Supplementary Tables S1 and S2 for detailed information on patients and microRNA results). Targetscan software was applied to identify down-regulated miRNAs that are putative regulator of HIF-1α gene and four miRNAs with robust binding sites in HIF-1α transcript, including miR-153, miR-543, miR-194 and miR-494, were identified (Fig. 1b). Previous studies found miR-153 expression was significantly down-regulated in drug-resistant K562 cells^21^ while miR-494 played a critical role in multidrug resistance in SW480 cells^22^. Accordingly, in the present study, we focused on the function of miR-153 and miR-494 in regulating HIF-1α expression and refractory epilepsy. Consistent with the microarray data, real-time PCR analysis in 32 surgical mTLE cases and 18 controls confirmed miR-153 and miR-494 were both down-regulated in temporal cortex of patients with mTLE (see Table 1 and Fig. 2 for patients information and PCR results).

### miR-153 is down-regulated in plasma of mTLE patients

**Figure 3:**
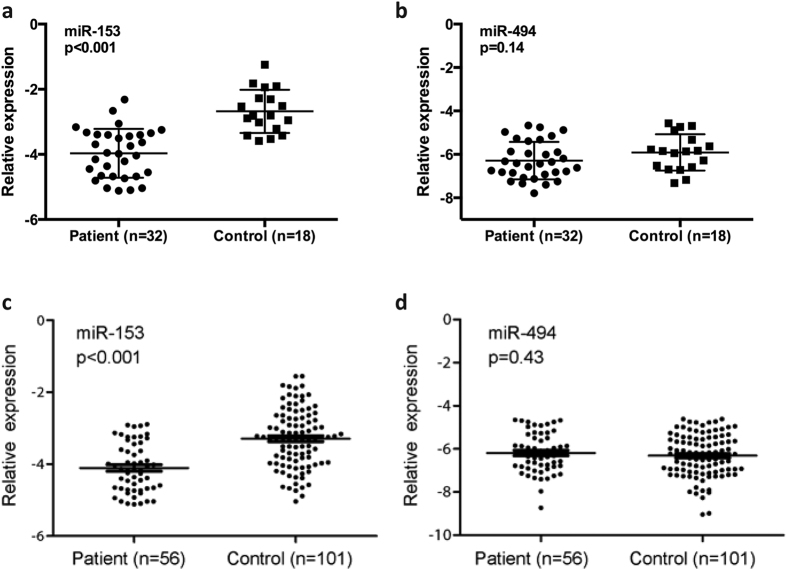
Relative expression of miR-153 in plasma of mTLE patients and controls. (**a**) miR-153 expression was examined by real-time quantitative PCR in the same 32 surgical patients with mTLE and 18 age matched surgical controls. (**b**) miR-494 expression in 32 surgical mTLE cases and 18 surgical controls. (**c**) miR-153 expression in overall 56 patients with mTLE and 101 healthy non-surgical controls. (**d**) miR-494 expression in 56 mTLE cases and 101 controls. Difference in miRNA expression was determined by student’s t-test.

Recent studies suggested miRNAs are highly stable in blood^23^,^24^. Consistent with data from brain tissue, we observed significantly reduced expression of miR-153 in plasma of 32 surgical patients with mTLE patients as compared with 18 surgical controls (p < 0.001) (Fig. 3a). In contrast, no significant difference in miR-494 expression was observed between these surgical patients and controls (p = 0.14) (Fig. 3b). Furthermore, down-regulation of miR-153 in plasma was validated in overall 56 mTLE patients with/without surgery compared to 101 healthy non-surgical controls (p < 0.001) (see Table 2 and Fig. 3c for patients information and PCR results), while no significant difference of plasma miR-494 level was found (p = 0.43) (Fig. 3d).

### Low plasma miR-153 level is a risk factor for mTLE

Multivariable linear regression analysis was performed to test whether reduced miR-153 in plasma is an independent risk factor associated with mTLE (Table 3). In a uni-variate linear regression model (model 1), we found miR-153 abundance is negatively correlated with mTLE in a significant manner (β = −0.26, p < 0.001). In a multi-variate linear regression model, after controlling for age and gender (model 2), low miR-153 level remained as an independent risk factor for mTLE (β = −0.25, p < 0.001). The results remained robust (β = −0.22, p < 0.001) even after considering all clinical characteristics such as Fasting glucose and Alanine aminotransferase (model 3).

### miR-153 overexpression decreases HIF-1α level in rat astrocytes

**Figure 4:**
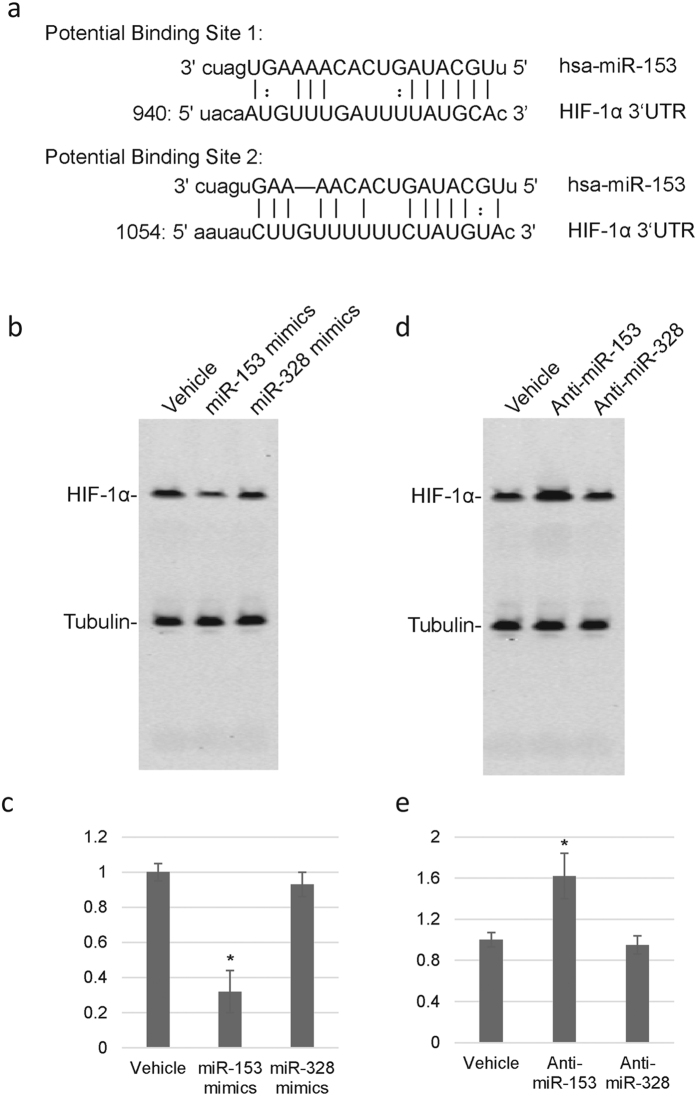
MiR-153 inhibits protein expression of HIF-1α. (**a**) Putative miR-153 binding sites predicted by targetscan in 3′-UTR of human HIF-1α gene. (**b,c**) Transfection of miR-153 mimics significantly reduces expression of HIF-1α in astrocytes as compared with control transfection with vehicle or miR-328 mimics. (**d,e**) Transfection of miR-153 inhibitor significantly enhanced expression of HIF-1α in astrocytes as compared with transfection with vehicle or miR-328 inhibitor.

Based on target prediction, there are two putative miR-153 binding sites in the 3′UTR of HIF-1α mRNA, localized between 944bp–964bp, and 1059bp–1075bp, respectively (Fig. 4a). Pharmacoresistant astrocytes cells were transfected with exogenous human miR-153 (miR-153 mimics) or a control miRNA with no putative biding site in HIF-1α (miR-328) or vehicle only as controls. As expected, overexpression of miR-153 dramatically inhibited protein expression of HIF-1α compared with mock or control transfections (Fig. 4b). In addition, we then inhibited miR-153 expression in astrocytes using anti-miR-153 morpholinos (MOs). MOs targeting miR-328 or vehicle only were used as controls. As expected, HIF-1α expression was significantly up-regulated as a result of miR-153 inhibition (Fig. 4c), but not in miR-328 inhibition group. Together, these results established that HIF-1α expression is repressed by miR-153.

### miR-153 directly targets HIF-1α 3′UTR

**Figure 5:**
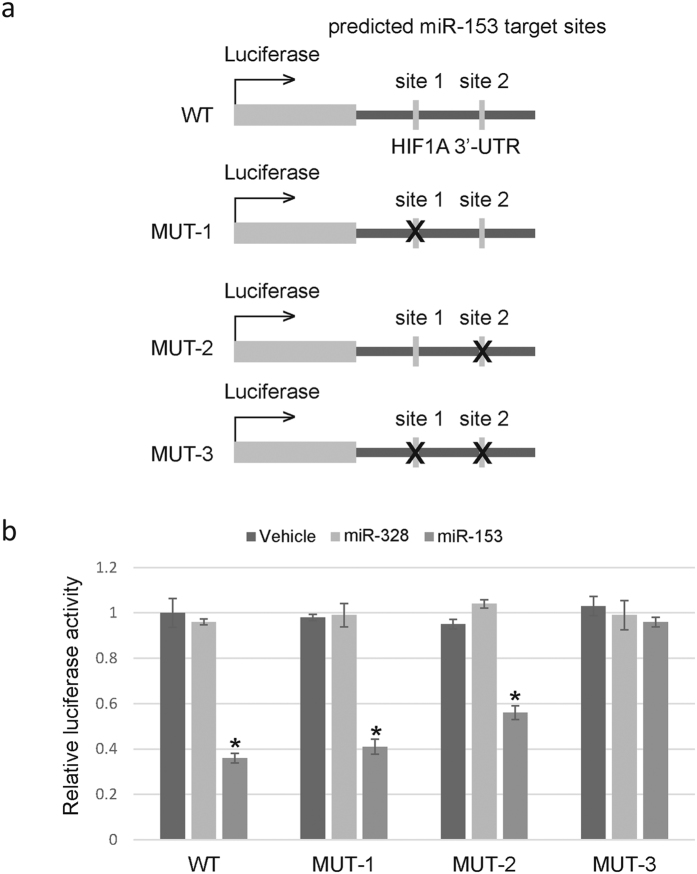
MiR-153 directly targets 3′-UTR of human HIF-1α gene. (**a**) Schema of luciferase reporter constructs used in the luciferase assays. A 217 fragment of HIF-1α 3′-UTR containing two possible miR-153 target sites predicted by TargetScan was inserted after the luciferase gene. The “X” sign over the gray boxes represents the deleted miRNA-binding sites. ‘WT’ stands for wild type and ‘MUT’ for mutant. (**b**) Dual luciferase assays of astrocytes cotransfected with miR-153 mimics and luciferase reporters containing WT or MUT HIF-1α 3′-UTR. Normalized firefly luciferase activity (firefly luciferase activity/Renillaluciferase activity) was calculated. Histograms show mean ± SD values of the relative luciferase activity of cells transfected with miR-153 with respect to those transfected with vehicle only or control miRNA. For each transfection, luciferase activity was averaged from six replicates. *P < 0.001, student’s t-test.

A 217bp fragment of HIF-1α 3′-UTR containing the two possible miR-153 target sites was cloned and inserted after the luciferase reporter gene (Fig. 5a, WT). We also constructed three mutant vectors with impaired miR-153 binding site 1, 2 or both, respectively (Fig. 5a, MUT-1~3). Of note, co-transfection of luciferase reporters and miR-153 mimics or control revealed that, miR-153 significantly decreased the relative luciferase activity of WT, MUT-1 and MUT-2, but had no effect on MUT-3 (Fig. 5b). These results supported the role of miR-153 in directly inhibiting HIF-1α expression, and suggested that both binding sites are essential for miR-153-induced inhibition of HIF-1α expression.

## Discussion

The key finding of our study is that miR-153 is significantly down-regulated in both temporal cortex tissue and plasma of patients with mTLE. Bioinformatics analysis predicted and our results determined that HIF-1α is a direct target of miR-153. Overexpression of miR-153 in rat astrocytes inhibit HIF-1α transcription while inhibition of miR-153 significantly up-regulated HIF-1α expression. These findings suggest that decreased miR-153 may play a critical role in drug-resistant epilepsy and may serve as a potential diagnostic marker and therapeutic target for refractory epilepsy.

Overexpression of multidrug resistance gene 1 (MDR1), specifically P-gp in brain astrocytes decreases accumulation of AEDs in the brain, which has been widely accepted as a crucial factor in drug-resistant epilepsy^25^,^26^. Numerous studies on tumor pharmacoresistance mechanism suggest MDR1 expression is modulated by HIF-1α^27^,^28^. In our previous study, we found HIF-1α and P-gp are coordinately overexpressed in hippocampus and temporal lobe of patients and animal models with refractory epilepsy^14^, which was again confirmed in this study. Activated HIF-1α is shown to enhance the transcription of P-gp in astrocytes, leading to lower concentration of AED in the brain^12^. Silencing HIF-1α by siRNA could reversely increase the sensitivity of transfected cells in different drug-resistant models^11^,^29^. Collectively, it suggests that HIF-1α may be a core factor involving drug-resistance of epilepsy and may be a potential therapeutic intervention strategy. However, the underlying mechanism of HIF-1α deregulation in epilepsy remains poorly understood.

MicroRNAs, a class of small noncoding RNA molecules that function as post-transcriptional regulators of gene expression by sequence complementarities with their target mRNA molecules, have been identified as key regulators in almost all aspects of cellular processes, such as cell proliferation, differentiation, apoptosis and cellular response to stimulus^30^. Recent studies support that aberrant alterations of miRNAs have been associated with a variety of neurological disorders, including epilepsy^17^,^31^. However, it still remains largely unknown how miRNAs contribute to multidrug resistance in epilepsy. In this study, we performed a genome-wide miRNA expression profiling and identified 65 down-regulated miRNAs and 29 up-regulated miRNAs in temporal cortex tissue isolated from patients with refractory mTLE. By targeting different mRNAs, these miRNAs might play important roles in fine-tuning of signaling pathways that control normal brain development and drug resistance in refractory epilepsy.

In this study, both miR-153 and miR-494 were found down-regulated in the temporal cortex of patients with mTLE and predicted as a putative regulator of HIF-1α gene with robust binding sites in HIF-1α transcript by bioinformatics analysis. However, only miR-153 was shown also decreased in the peripheral plasma. Similarly, previous literature reported dysregulation of miR-494 in pathogenesis of epilepsy in both animal models and clinical studies^32^. However, to the best of our knowledge, this is the first work that shows decreased miR-153 level is related to drug-resistant epilepsy through mediating overexpression of HIF-1α. The discrepancies with previous studies may due to different inclusion criteria and standard for the surgery selection of patients with mTLE and varied technics and standards applied to screen for significantly dysregulated miRNAs. In addition, application of different animal models and/or brain regions, extraneous effects such as racial, regional difference and other individual characteristics may also affect the profiling of miRNA abundance. Thus, the results required to be further validated in the future.

Using miRNA overexpression, miRNA function inhibition and luciferase assay, we demonstrated that overexpression of miR-153 resulted in up-regulation of HIF-1α, which is immediately linked to pharmacoresistance via inducing expression of numerous drug transporters and decreasing accumulation of AEDs in the brain. Furthermore, inhibition of miR-153 significantly decreased the HIF-1α expression. Although gene expression is regulated at multiple transcriptional and post-transcriptional levels^33^,^34^,^35^,^36^,^37^,^38^, our data provides clear evidence that miR-153 is a critical regulator of HIF-1α expression and may serve as a potential therapeutic strategy for drug-resistant epilepsy.

miRNAs might easily penetrate the blood–brain barrier (BBB) and are highly stable in blood. Several previous studies confirmed selected miRNAs expression in injured brain tissues and blood are highly correlated^39^. In line with results from brain tissue, we found that abundance of plasma miR-153 is significantly reduced in the same surgical patients as well as a larger cohort of mTLE patients as compared with controls. On the contrary, plasma miR-494 showed no difference between patients and controls. Multivariable linear regression analysis showed the reduced miR-153 in plasma is an independent risk factor associated with mTLE, suggesting miR-153 might be used as a robust biomarker for drug-resistant epilepsy.

Of note, in addition to its putative role in regulating HIF-1α expression and drug resistance, recent data support a major role for miR-153 in fine-tuning of signaling pathways that control synaptic transmission and neuronal development^40^. The discovery of several brain-specific miRNAs, such as miR-9, miR-124a, miR-124b, miR-135, miR-153, miR-183, and miR-219, in mouse and human differentiating neurons suggested these miRNAs as effectors in mammalian neuronal processes^41^. Interestingly, previous microarray profiling has identified miR-153 as one of the most significantly decreased miRNA in fetal neural stem cells (NSCs) following ethanol exposure, supporting its role in protecting against teratogenic effects of chemical exposure in neural cells^42^. In a study by Doxakis *et al.*, researchers also found down-regulation of miR-153 might lead to alpha-synuclein accumulation, which contributes to the pathogenesis of Parkinson disease^43^. As suggested previously, each miRNA can potentially regulate hundreds of target genes^44^, with the prediction that more than one-third of all human genes may be regulated by miRNAs^45^. Further studies are needed to explore other functions of miR-153 in epileptogenesis and drug-resistance using both patient samples and epilepsy animal models.

There are several limitations of our study. Firstly, the number of brain samples used to validate the microRNA profiling is relatively limited and no hippocampus tissue was detected due to difficulty in tissue achieving especially the normal hippocampus as controls. Secondly, although we demonstrate miR-153 can regulate HIF-1α in rat astrocytes *in vitro*, animal models of mTLE should be used in the future to further confirm our findings. Thirdly, as we have mainly focused on miR-153 in this study, the role of other deregulated miRNAs, such as miR-543 and miR-494, in the epileptogenesis and/or drug-resistance should be further investigated in the future.

In conclusion, our results suggest that miR-153 is significantly down-regulated in temporal cortex and plasma of patients with refractory epilepsy. This might be linked to up-regulation of HIF-1α in the patients’ brain tissue and provide a promising therapeutic target for refractory epilepsy. As increasing number of miRNAs in regulating pathogenesis of epilepsy have been discovered, we suggest that this connection may be widespread^42^. Considering the fact that different brain subfields may function differentially and have various contributions to the establishment of an epileptic state, future studies with detailed miRNA profiling in each structure would certainly facilitate data interpretation, and lead to the identification of genetic subtypes and their association with treatment response.

## Methods

### mTLE patients and control group

56 patients who were diagnosed as mTLE and had undergone treatment in West China Hospital from Jan 2010 to Jan 2013 were included in this study. 32 of 56 patients with mTLE had undergone anterior temporal lobectomy in our hospital. All patients were comprehensively evaluated and met the definition of mTLE^46^: (I) seizure semiology consistent with mTLE, usually with epigastric/autonomic/psychic auras, followed by complex partial seizures; (II) electroencephalography confirmed the seizure onset zone in the temporal lobe; (III) Magnetic resonance imaging showed no lesions other than uni- or bilateral hippocampal atrophy (reduced hippocampal dimensions and increased T2 signal); (IV) clinical histopathological examination consistent with hippocampus sclerosis; and (V) no evidence of dual pathology identified by any assessment. In addition, 18 patients who had undergone surgical treatment for head trauma or cerebral hemorrhage and 101 age-matched healthy controls were also included.

The study was approved by the Ethics Committees of West China Hospital. Informed consent was obtained from each patient and all experiments were performed in accordance with relevant guidelines.

### Brain tissue collection and RNA extraction

Temporal cortex tissues resected from 32 patients with refractory mTLE who had undergone anterior temporal lobectomy were collected. The 18 control samples of temporal neocortical tissues without abnormal pathological changes were obtained from neurosurgery department of the same hospital. The control group included 5 head trauma and 13 cerebral hemorrhage cases. None of patients in control group was ever diagnosed of epilepsy or seizures. Resected brain tissues were immediately minced to small pieces and frozen in liquid nitrogen for further studies. For miRNA expression analysis, small RNAs from temporal cortex tissue from patients or controls were isolated using miRNA extraction kit (Qiagen) according to manufacture’s instruction. For detection of HIF-1α mRNA in brain tissue, RNA was extracted according to Trizol method (Invitrogen, USA).

### Plasma collection and RNA Extraction

5 ml blood samples from all 56 mTLE cases, 18 surgical control cases and 101 healthy controls were collected in EDTA-containing tubes. Whole-blood samples were centrifuged at 1800 g for 20 min at 4 °C after blood collection, and the upper plasma phase was carefully transferred into microcentrifuge tube. A second centrifugation at 1600 g for 10 min at 4 °C to remove additional cellular debris and minimize contamination of cell-free nucleic acids derived from damaged blood cells. For long storage, plasma frozen in aliquots was kept at −80 °C until further analysis. Total RNA was extracted from 100 μl of plasma samples using TRIzol LS reagent (Life Technologies) according to manufacturer’s instructions. 3 μl synthetic Caenorhabditis elegans miRNA cel-miR-39 (5′-UCACCGGGUGUAAAUCAGCUUG-3′, 10 nM) was used as spike-in control and added directly to each sample. RNA was re-suspended in 30 μl of nuclease-free water and tested using a NanoDrop ND-1000 spectrophotometer (Thermo Scientific).

### MicroRNA expression profiling

The GeneChip miRNA 1.0 array (Affymetrix) was used for the miRNA expression profiling in 5 patients with refractory mTLE who had undergone epileptic surgery and 3 age-matched surgical controls. Equal amount of small RNAs isolated from different patients or controls were mixed to generate a pool of patient and a pool of control. 1 μg of small RNAs in each pool were used for microarray analysis using GeneChip miRNA Array. Array hybridization, washing, and scanning of the slides were carried out by Beijin Capitalbio Corporation, inc. Microarray raw data were analyzed using the GeneSpring software (Agilent). Signals from different microarrays were normalized by mean gene expression value. Fold change >1.5 was used as a cut-off to identify up- and down-regulated miRNAs between different groups. We used online tools Targetscan (http://www.targetscan.org) for target prediction.

### Real-time quantitative PRC

For detection of HIF-1α in brain tissue, PCR primers were designed and synthesized by Shanghai Shenggong (China). The β-actin was taken as an internal control. RT-qPCR was performed with the FTC2000 PCR system (Funglyn, Canada). The following conditions were used for amplification: 94 °C for 2 min; 94 °C for 20 s; 50 °C for 20 s; and 60 °C for 30 s for 45 cycles. The relative expression levels were calculated by using the ΔCt method^47^. For detection of miR-153 in brain/plasma sample, 10 μl purified RNA was reverse transcribed using mature miRNA-specific primers and miScript II RT Kit (Qiagen) following the manufacturer’s protocol. The q-PCR assay was carried out in a 10 μl reaction mixture using miScript SYBR Green PCR Kit (Qiagen) and the following thermal cycling condition: 95 °C for 10 min, 40 cycles of PCR amplification at 95 °C for 15 s, 60 °C for 1 min. U6 and cel-miR-39 were used as internal controls to normalize miRNA in brain and plasma, respectively. The relative miRNA expression in each individual sample calculated by ΔCt method.

### Rat astrocyte culture and pharmacoresistant astrocyte model establishment

Rat astrocytes were isolated as described in our previous study^48^ and were identified by immunocytochemical staining with glial fibrillary acidic protein (GFAP). All procedures were carried out in accordance with the Chinese Animal Welfare Act and approved by the responsible governmental agency at Sichuan University.

Pharmacoresistant astrocyte model was built as described in our previous study^48^. Briefly, coriaria lactone (CL), a compound extracted from the plant loranthus or coriaria sinica maxim which can incur epilepsy in animals, was provided by the Pharmaceutical Company of Sichuan University (coriaria lactone injection ampoules, 1 ml = 55 mg; lot no. 980715) and was added to medium (12 ml/ml) to induce rat astrocytes to express HIF-1α. After 12 days induction, rat astrocytes grown under these conditions had high P-gp over-expression and optimal cell morphology were used in subsequent experiments.

### Transfection

miRNA mimics or morpholino inhibitors for hsa-miR-153 (Ambion, Austin, TX) at a concentration of 50 nM were incubated with Lipofectamine 2000 (Invitrogen) in culture medium before addition to cells according to the manufacturer’s protocol. Vehicle treatment only and miRNA mimics or morpholino inhibitors for hsa-miR-328 (not targeting HIF-1α) were incubated under the same condition as controls.

### Luciferase reporter assay

A 217 fragment of HIF1A 3′-UTR containing two possible miR-153 target sites predicted by TargetScan was cloned using primers: F: 5′-TAGCAAAATTGCCTAGTAT-3′, R: 5′-TAGATCCAACCACAAAGAG-3′, and subcloned into TA-vector (Life science). The fragment then subcloned into pmirGLO dual-luciferase reporter vector (Promega,) using bamH I and Xho I restriction enzymes (NEB). The reporter gene constructs were cotransfected into cells containing a miR mimic control or miR-153 mimic for 36 h. The dual luciferase system (Promega) was used to measure luciferase activity following manufacturer’s protocol. Normalized firefly luciferase activity (firefly luciferase activity/Renilla luciferase activity) was used to compare the expression of miR-153 between different samples. For each transfection, luciferase activity was averaged from three replicates. Mutations were generated using the QuikChange Site-Directed Mutagenesis Kit (Agilent). For MUT-1, the WT sequence of binding site 1 TACAATGTTTGATTTT was changed to the antisense sequence AAAATCAAACATTGTA that impairs miRNA binding; For MUT-2, the WT sequence of binding site 2 AATATCTTGTTTTTTCTATGTAC was changed to the antisense sequence GTACATAGAAAAACAAGATATT.

### Statistical analyses

All statistic analyses were performed by SPSS software 18.0 (SPSS, Inc., Chicago, USA). Relative expression of miRNAs was calculated by using the fold change in miRNAs expression method. Differences in miRNAs expression were determined by student’s t-test. We used ANOVA, multiple logistic regression analysis and ROC analysis to evaluate the diagnostic potential of different plasma miRNAs and its association with different physiological characteristics. P value < 0.05 (two-tailed) was considered statistically significant.

## Additional Information

**How to cite this article**: Li, Y. *et al.* Aberrant expression of miR-153 is associated with overexpression of hypoxia-inducible factor-1α in refractory epilepsy. *Sci. Rep.* **6**, 32091; doi: 10.1038/srep32091 (2016).

## Supplementary Material

Supplementary Information

## Figures and Tables

**Table 1:** Clinical information of 32 surgical patients with mTLE and 18 controls included for analysis of plasma miR-153 level.

	mTLE group	Controls
Number	32	18
Gender (M/F)	19/13	12/6
Mean age (years)	24.94 ± 11.73	27.56 ± 12.46
Epilepsy duration	11.50 ± 5.20	—
Seizure type		
CPS	3 (9.4%)	—
sGTCS	11 (34.4%)	—
SPS/CPS + sGTCS	18 (56.3%)	—
AEDs intake		
Two AEDs	9 (28.1%)	—
Three AEDs	20 (62.5%)	—
Four or more AEDs	3 (9.4%)	—

The control group includes 5 cases with head trauma and 13 cases with cerebral hemorrhage. SPS: simple partial seizure; CPS: complex partial seizure; sGTCS: secondary generalized tonic-clonic seizure; AEDs: anti-epileptic drugs. AEDs used by patients include: phenobarbitone, phenothiazine, carbamazepine, lamotrigine, levetiracetam, valproate acid, topiramate, oxcarbazepine and clonazepam.

**Table 2:** Clinical information of overall 56 patients with mTLE and 101 healthy controls included for analysis of plasma miR-153 level.

	mTLE group	Controls
Number	56	101
Gender (M/F)	30/26	57/44
Mean age (years)	23.3 ± 10.8 (7−62)	27.5 ± 11.2 (6–65)
Epilepsy duration	10.3 ± 4.9 (3–25)	—
Seizure type		
SPS	1 (1.8%)	—
CPS	6 (10.7%)	—
sGTCS	20 (35.7%)	—
SPS/CPS + sGTCS	29 (51.8%)	—
AEDs intake		
Two AEDs	15 (26.8%)	—
Three AEDs	35 (62.5%)	—
Four or more AEDs	6 (10.7%)	—

SPS: simple partial seizure; CPS: complex partial seizure; sGTCS: secondary generalized tonic-clonic seizure; AEDs: anti-epileptic drugs. AEDs used by patients include: phenobarbitone, phenothiazine, carbamazepine, lamotrigine, levetiracetam, valproate acid, topiramate, oxcarbazepine and clonazepam.

**Table 3:** Multiple linear regression analyses of the association between miR-153 and mTLE.

	Model 1	Model 2	Model 3
miR153 abundance (−ΔCT)	−0.26 (<0.001)	−0.25 (<0.001)	−0.22 (<0.001)
Gender (male/female)		−0.06 (0.40)	−0.06 (0.42)
Age (years)		0.00 (0.21)	0.00 (0.81)
Fasting glucose (mmol/l)			−0.11 (0.06)
Alanine aminotransferase (U/L)			0.00 (0.08)
Serum creatinine (mmol/l)			0.00 (0.63)
Blood urea nitrogen (mmol/L)			−0.04 (0.12)
Uric acid (mg/dL)			0.00 (0.58)
Total cholesterol (mg/dL)			−0.06 (0.37)
Triglyceride (mg/dL)			0.05 (0.41)
Low-density lipoprotein (mg/dL)			0.02 (0.85)
High-density lipoprotein (mg/dL)			0.03 (0.77)

Model 1: unadjusted; Model 2: adjusted for gender and age; Model 3: adjusted for all Baseline characteristics examined. Association between different variables and epilepsy are shown as Standard β (p-value).
