# Supplementary material for: Aberrant expression of miR-153 is associated with overexpression of hypoxia-inducible factor-1α in refractory epilepsy
Source: Sci Rep. 2016 Aug 24;6:32091. doi: 10.1038/srep32091 (PMC4995460; doi:10.1038/srep32091)
Supplement: Supplementary Information [file srep32091-s1.doc]

**Aberrant expression of miR-153 is associated with overexpression of hypoxia-inducible factor-1α in refractory epilepsy**

Yaohua Li^1, #^, MD, Cheng Huang^1, #^, MD, Peimin Feng^2, #^, MD, Yanping Jiang^1^, MD, Wei Wang^1^, MD, Dong Zhou^1^, MD, Lei Chen^1^, *, MD

Supplementary Table S1. Clinical information of 5 patients with mTLE and 3 controls for mircroarray analysis.

| **ID** | **Gender** | **Age (years)** | **Epilepsy duration (years)** | **Seizure type** | **Side** | **Antiepileptic drugs** |
| --- | --- | --- | --- | --- | --- | --- |
| **mTLE 1** | Male | 28 | 9 | CPS + GTCS | L | CBZ, LEV, VPA |
| **mTLE 2** | Male | 30 | 14 | CPS + GTCS | L | PB, CBZ, VPA |
| **mTLE 3** | Male | 23 | 7 | CPS +GTCS | R | VPA, OXC, LEV |
| **mTLE 4** | Female | 20 | 13 | CPS | L | VPA, CBZ, TOP |
| **mTLE 5** | Male | 32 | 11 | CPS + GTCS | R | VAP, OXC, LTG |
| **ID** | **Gender** | **Age (years)** | **-** | **-** | **Side** | **Diagnosis** |
| **Control 1** | Male | 38 | - | - | R | Cerebral hemorrhage |
| **Control 2** | Male | 28 | - | - | L | Cerebral hemorrhage |
| **Control 3** | Female | 23 | - | - | L | Cerebral hemorrhage |

mTLE, mesial temporal lobe epilepsy; R, right side; L, left side; CBZ, carbamazepine; LEV, levetiracetam; VPA, valproate acid; PB, phenobarbitone; OXC, oxcarbazepine; TOP, topiramate; LTG, lamotrigine.

Supplementary Table S2. Differently expressed miRNA in epilepsy patients compared with controls (Fold change>1.5).

| **Transcript ID** | **Patient expression** | **Patient call** | **Control expression** | **Control call** | **Fold change** |
| --- | --- | --- | --- | --- | --- |
| **mir-885** | 245.50 | P | 659.96 | P | 0.34 |
| **mir-1973** | 52.51 | P | 131.61 | P | 0.36 |
| **mir-146b** | 75.84 | P | 173.69 | P | 0.39 |
| **mir-411** | 85.37 | P | 185.29 | P | 0.42 |
| **mir-4298** | 94.47 | P | 201.20 | P | 0.42 |
| **mir-1296** | 92.88 | P | 179.91 | P | 0.47 |
| **mir-495** | 86.04 | P | 164.44 | P | 0.47 |
| **miR-153** | 47.32 | P | 90.30 | P | 0.47 |
| **mir-1226** | 13.49 | A | 25.70 | P | 0.47 |
| **mir-1225** | 88.88 | P | 168.07 | P | 0.48 |
| **mir-543** | 79.00 | P | 147.48 | P | 0.48 |
| **mir-504** | 116.09 | P | 215.93 | P | 0.49 |
| **mir-126** | 953.64 | P | 1765.94 | P | 0.49 |
| **mir-299** | 56.00 | P | 103.49 | P | 0.49 |
| **mir-935** | 362.12 | P | 662.02 | P | 0.49 |
| **miR-128** | 1518.18 | P | 2730.30 | P | 0.50 |
| **miR-30c** | 747.35 | P | 1314.12 | P | 0.51 |
| **mir-769** | 348.19 | P | 611.04 | P | 0.51 |
| **mir-220a** | 12.01 | A | 20.62 | P | 0.53 |
| **mir-342** | 121.86 | P | 208.95 | P | 0.53 |
| **mir-29a** | 963.75 | P | 1644.00 | P | 0.53 |
| **mir-187** | 98.18 | P | 165.44 | P | 0.54 |
| **mir-592** | 15.41 | A | 25.66 | P | 0.54 |
| **mir-628** | 157.26 | P | 261.13 | P | 0.54 |
| **mir-766** | 51.30 | P | 84.70 | P | 0.55 |
| **mir-3139** | 13.31 | A | 21.92 | P | 0.55 |
| **mir-125a** | 2563.83 | P | 4193.73 | P | 0.55 |
| **mir-323b** | 47.38 | P | 77.25 | P | 0.55 |
| **mir-3185** | 175.17 | P | 282.67 | P | 0.56 |
| **mir-331** | 174.51 | P | 281.15 | P | 0.56 |
| **mir-328** | 229.00 | P | 367.39 | P | 0.56 |
| **miR-194** | 80.54 | P | 126.21 | P | 0.58 |
| **mir-323** | 88.89 | P | 137.15 | P | 0.58 |
| **miR-3160** | 12.69 | A | 19.56 | P | 0.59 |
| **mir-212** | 280.26 | P | 431.47 | P | 0.59 |
| **mir-668** | 44.84 | P | 68.33 | P | 0.59 |
| **mir-485** | 93.76 | P | 141.78 | P | 0.60 |
| **miR-129-5p** | 307.93 | P | 463.27 | P | 0.60 |
| **mir-1231** | 34.78 | P | 51.66 | P | 0.61 |
| **mir-425** | 562.36 | P | 833.27 | P | 0.61 |
| **mir-383** | 295.40 | P | 436.82 | P | 0.61 |
| **mir-181d** | 411.37 | P | 607.48 | P | 0.61 |
| **mir-1224** | 37.48 | P | 54.91 | P | 0.62 |
| **mir-671** | 58.06 | P | 85.01 | P | 0.62 |
| **mir-29c** | 108.12 | P | 158.13 | P | 0.62 |
| **mir-149** | 1811.08 | P | 2618.33 | P | 0.62 |
| **mir-3154** | 23.04 | P | 33.09 | P | 0.63 |
| **mir-520c** | 14.60 | A | 20.88 | P | 0.63 |
| **mir-494** | 257.59 | P | 366.14 | P | 0.63 |
| **mir-4284** | 474.71 | P | 663.33 | P | 0.65 |
| **mir-647** | 15.86 | A | 22.15 | P | 0.65 |
| **mir-30b** | 259.34 | P | 361.87 | P | 0.65 |
| **mir-222** | 2100.06 | P | 2925.95 | P | 0.65 |
| **mir-323b** | 17.03 | A | 23.65 | P | 0.65 |
| **mir-378c** | 120.83 | P | 166.97 | P | 0.65 |
| **mir-1180** | 510.12 | P | 704.08 | P | 0.65 |
| **miR-329** | 116.52 | P | 160.75 | P | 0.65 |
| **mir-487a** | 208.30 | P | 285.35 | P | 0.66 |
| **miR-129-3p** | 258.63 | P | 353.57 | P | 0.66 |
| **mir-139** | 2238.61 | P | 3059.09 | P | 0.66 |
| **mir-95** | 26.17 | P | 35.74 | P | 0.66 |
| **mir-154** | 81.62 | P | 110.83 | P | 0.66 |
| **mir-922** | 16.24 | A | 22.04 | P | 0.67 |
| **mir-382** | 578.04 | P | 782.70 | P | 0.67 |
| **mir-486** | 308.36 | P | 185.41 | P | 1.50 |
| **mir-374b** | 37.91 | P | 22.64 | P | 1.51 |
| **mir-320a** | 3289.26 | P | 1948.69 | P | 1.52 |
| **mir-21** | 45.31 | P | 26.44 | P | 1.55 |
| **mir-1250** | 46.02 | P | 26.37 | P | 1.57 |
| **mir-455** | 30.33 | P | 16.85 | P | 1.62 |
| **mir-886** | 133.89 | P | 74.32 | P | 1.63 |
| **mir-4324** | 94.33 | P | 51.29 | P | 1.66 |
| **mir-1287** | 55.22 | P | 29.58 | P | 1.68 |
| **mir-887** | 70.93 | P | 37.74 | P | 1.70 |
| **mir-500** | 110.54 | P | 58.54 | P | 1.70 |
| **mir-574** | 587.10 | P | 305.37 | P | 1.73 |
| **miR-92a** | 1970.20 | P | 1004.40 | P | 1.77 |
| **mir-193a** | 151.56 | P | 76.96 | P | 1.78 |
| **mir-152** | 258.09 | P | 128.27 | P | 1.82 |
| **mir-338** | 59.98 | P | 29.78 | P | 1.82 |
| **mir-886** | 75.25 | P | 36.90 | P | 1.84 |
| **mir-92b** | 1026.04 | P | 498.05 | P | 1.86 |
| **mir-455** | 401.58 | P | 180.35 | P | 2.01 |
| **mir-584** | 176.42 | P | 76.66 | P | 2.08 |
| **mir-3195** | 39.14 | P | 16.89 | A | 2.09 |
| **mir-933** | 90.00 | P | 37.58 | P | 2.16 |
| **mir-1274a** | 77.40 | P | 32.00 | P | 2.18 |
| **mir-1246** | 51.10 | P | 19.14 | A | 2.41 |
| **let-7b** | 28280.06 | P | 9512.42 | P | 2.68 |
| **mir-451** | 177.27 | P | 58.03 | P | 2.76 |
| **mir-219-2** | 1876.39 | P | 594.04 | P | 2.85 |
| **mir-34c** | 148.26 | P | 24.72 | P | 5.41 |
| **mir-34c** | 372.17 | P | 21.49 | A | 15.63 |
| **mir-184** | 925.22 | P | 41.46 | P | 20.14 |
